# Absorbing filter AN69 surface treatment in critically ill COVID-19 patients: a single-center experience

**DOI:** 10.1080/0886022X.2021.1997763

**Published:** 2021-11-09

**Authors:** Gang Chen, Jie Ma, Peng Xia, Yan Hu, Zhengyin Liu, Xiang Zhou, Taisheng Li, Xiaowei Yan, Limeng Chen, Xuemei Li, Yan Qin, Shuyang Zhang

**Affiliations:** Department of Nephrology, Peking Union Medical College Hospital, Chinese Academy of Medical Sciences, Beijing, China; Department of Infectious Disease, Peking Union Medical College Hospital, Chinese Academy of Medical Sciences, Beijing, China; Department of Intesive Care Medicine, Peking Union Medical College Hospital, Chinese Academy of Medical Sciences, Beijing, China; Department of Cardiology, Peking Union Medical College Hospital, Chinese Academy of Medical Sciences, Beijing, China

Dear Editors,

More than 20% of patients with coronavirus disease 2019 (COVID-19) in the intensive care unit (ICU) developed acute kidney injury (AKI) and worsened clinical outcomes [1]. Cytokine storm syndrome (CSS), featured by an overactive immune response and unsustainable cytokine release, is suspected in the COVID-19 [2]. The rationale for extracorporeal support with blood purification strategies partially targets CSS and has been practiced in severe COVID-19 patients [3,4]. A randomized controlled trial (RCT) indicated that the addition of therapeutic plasma exchange (TPE) to standard ICU therapy was associated with faster clinical recovery, but without a significantly lower 35-day mortality [5].

The AN69 membrane is featured with high-throughput materials with high adsorption levels, improving the removal of medium to high molecular weight solutes through ionic charge interactions. The AN69 surface treatment (AN69ST) has also been attempted in severe COVID-19 cases. However, current evidence of AN69ST in COVID-19 is limited to case series and observational studies, which demonstrated potential therapeutic roles in decreasing cytokine levels, stabilizing hemodynamic status, improving multiple organ dysfunction syndromes (MODS) and mortality rate [6–8]. Despite the lack of RCT evidence, the US Food and Drug Administration (FDA) recommends immuno-adsorption for emergency use in critically ill patients with COVID-19 [9].

Here we presented a series of 12 critically ill COVID-19 patients in China, characterized with profound inflammation and applied with modified AN69ST (oXiris). All patients were included from February to April 2020. The AN69 filters were renewed every 12–24 h for 3–7 treatment sessions until satisfactory inflammatory factor removal or severe complications were considered. We further compared the demographic characteristics, clinical manifestations, laboratory data, and medications in survived and non-survived patients based on the prognosis.

Three out of the twelve patients survived after AN69ST. Among all 12 patients, the average age was 67.83 ± 7.59 years, and seven were males. Some chronic comorbidities, such as hypertension, coronary artery disease, cerebral infarct, diabetes, and chronic obstructive pulmonary disease, were reported in 58.3%, 16.7%, 16.7%, 8.3%, and 8.3% of patients representatively. The mean duration from disease onset to admission was 12.42 ± 5.00 days. There was no difference in sex, age, background diseases, and time from disease onset to admission between the survived and non-survived groups (Table 1).

**Table 1.** Clinical characteristics of patients with severe COVID-19 underwent AN69ST.

Characteristics	Total (*n*%)	Survived	non-survived	*p* (Survived vs. non-survived)
Male, n(%)	7/12 (58.3)	2/3 (66.7)	5/9 (55.6)	>0.999
Age, year	67.83 ± 7.59	65.67 ± 8.51	68.56 ± 7.67	0.593
Background Disease, n(%)				
Hypertension	7/12 (58.3)	2/3 (66.7)	5/9 (55.6)	>0.999
Diabetes	1/12 (8.3)	1/3 (33.3)	0/9 (0.0)	0.250
CAD	2/12 (16.7)	1/3 (33.3)	1/9 (11.1)	0.455
Cerebal infarct	2/12 (16.7)	1/3 (33.3)	1/9 (11.1)	0.455
COPD	1/12 (8.3)	0/3 (0.0)	1/9 (11.1)	>0.999
Onset to admission, day	12.42 ± 5.00	13.67 ± 4.93	12.00 ± 5.24	0.640
In-hospital length, day	30.58 ± 12.03	35.67 ± 12.34	28.89 ± 12.17	0.424
ICU length, day	20.17 ± 9.98	20.33 ± 11.02	20.11 ± 10.33	0.975
Incubation length, day	21.67 ± 11.17	19.00 ± 9.64	22.56 ± 12.03	0.655
Time to AN69ST, day	19.25 ± 11.97	18.33 ± 8.15	19.56 ± 13.42	0.887
Treatments, n(%)				
Glucocorticoid	11/12 (91.7)	3/3 (100.0)	8/9 (88.9)	>0.999
Antiviral	9/12 (75.0)	2/3 (66.7)	7/9 (77.8)	>0.999
IVIG	12/12 (100.0)	3/3 (100.0)	9/9 (100.0)	NA
Anticoagulation	9/12 (75.0)	3/3 (100.0)	6/9 (66.7)	0.509
Invasive ventilation	12/12 (100.0)	3/3 (100.0)	9/9(100.0)	NA
ECMO	4/12 (33.3)	1/3 (33.3)	3/9 (33.3)	>0.999

COVID-19: coronavirus infection disease 2019; AN69ST: AN69 surface treatment; CAD: coronary artery disease. COPD: chronic obstructive pulmonary disease; ICU: intensive care unit; IVIG: intravenous immunoglobulin; ECMO: extracorporeal membrane oxygenation.

The length of hospitalization was averaged 30.58 ± 12.03 days, and the ICU stay was 20.17 ± 9.98 days. Glucocorticoid (91.7%), antiviral agents (75.0%), intravenous immunoglobulin (IVIG) (100.0%), and anticoagulation (75.0%) were administrated in patients during their ICU stay, and there was no difference between survived and non-survived groups. All patients received invasive ventilation. Extracorporeal membrane oxygenation (ECMO) was equally performed in 33.3% of patients for both groups (Table 1).

All patients demonstrated significant lymphocytopenia, with an average lymphocyte count of 0.57 ± 0.31 × 10^9^/L. We noticed the increased inflammatory factors in these critical patients, including hypersensitive C reactive protein (hs-CRP), interleukin (IL)-2 receptor, IL-6, IL-8, IL-10, tumor necrosis factor (TNF)-α, and Ferrin. Patients who survived were inclined to show lower inflammatory factors but without significance compared with the non-survived group. Biomarkers referred to cardiac injury, including troponin I and creatinine kinase MB, concerningly increased in both groups. On ICU admission, patients who survived indicated a relatively more preserved kidney function in urea, serum creatinine, and cystatin C. However, statistics yielded no significant difference (Table 2).

**Table 2.** Laboratory measurements at ICU admission in severe COVID-19 patients underwent AN69ST.

Measurements	Total	Survived	non-survived	*p* (Survived vs. non-survived)
WBC (×10^9^/L)	12.96 ± 5.29	17.22 ± 6.57	11.54 ± 4.31	0.110
Lymphocyte (×10^9^/L)	0.57 ± 0.31	0.74 ± 0.49	0.52 ± 0.24	0.221
Hemoglubin (g/L)	129.17 ± 24.18	135.00 ± 7.81	127.22 ± 27.77	0.652
Platelet (×10^9^/L)	156.92 ± 91.39	166.00 ± 98.14	153.89 ± 95.56	0.853
Albumin (g/L)	29.75 ± 4.77	31.33 ± 2.25	29.22 ± 5.36	0.533
hsCRP (mg/L)	134.33 ± 77.73	119.20 ± 87.78	139.38 ± 79.16	0.746
IL-2R (U/mL)	1459.17 ± 995.18	817.33 ± 1017.39	1673.11 ± 947.12	0.283
IL-6 (pg/mL)	382.45 ± 904.96	68.43 ± 38.61	383.75 ± 703.01	0.469
IL-8 (pg/mL)	87.42 ± 106.90	36.90 ± 33.53	104.26 ± 118.98	0.369
IL-10 (pg/mL)	25.98 ± 36.18	9.27 ± 4.49	31.54 ± 40.68	0.381
TNF-α (pg/mL)	30.12 ± 47.00	8.43 ± 2.42	37.34 ± 52.91	0.381
Ferrin (mg/L)	2.75 ± 4.56	1.14 ± 0.57	3.11 ± 5.02	0.607
cTNI (pg/mL)	85.59 ± 154.42	191.23 ± 311.32	50.38 ± 54.57	0.516
CKMB (ng/mL)	7.03 ± 8.72	2.30 ± 1.01	8.61 ± 9.65	0.088
Urea (mmol/L)	10.64 ± 7.79	7.03 ± 3.17	11.84 ± 8.63	0.380
Creatinine (μmol/L)	111.25 ± 108.05	62.67 ± 30.92	127.44 ± 120.97	0.394
Cystatin C (mg/L)	1.39 ± 1.02	0.79 ± 0.68	1.62 ± 1.07	0.247

COVID-19: coronavirus infection disease 2019; AN69ST: AN69 surface treatment; WBC: white blood cell; hsCRP: hypersensitive C reactive protein; IL: interleukin; TNF: tumor necrosis factor; cTNI: cardiac troponin I; CKMB: creatine kinase MB.

The average time from admission to AN69ST was 19.25 ± 11.97 days; still, no significant difference appeared between survivors and non-survivors. The important candidate values to demonstrate the effects of AN69ST, such as IL-6, hs-CRP, and lymphocyte counts, ranged widely in our patients. Therefore, we performed log transformation for these data sets in further analysis. Figure 1 described the changes in IL-6, lymphocyte counts, and hs-CRP for individual patients during AN69ST. The log IL-6 readings demonstrated a decline for each patient after AN69ST. Survived patients generally appeared lower IL-6 than the non-survived group, and the IL-6 values quickly dropped to low levels after the first session of AN69ST. For most patients, the log lymphocyte counts inclined to increase after AN69ST. The manifestation of log hs-CRP values varied but demonstrated a decline in survived patients. Figure 2 demonstrated the overall change of IL-6 in both groups. The IL-6 values were not significantly different between the survived and non-survived groups before AN69ST. However, IL-6 responded differently to AN69ST in two groups. We found no significant IL-6 decrease after the first two sessions of AN69ST in the non-survived group, whereas IL-6 reduced significantly in the survived group after the first session of AN69ST (*p=* 0.027) and maintained lower levels. Before the survived group was transferred out of ICU, the last measurements of their IL-6 remained significantly lower than the measurements before AN69ST (*p=* 0.010). However, the last measure of IL-6 significantly raised in the non-survived group, compared with the initial values (pre-AN69ST vs. before death, *p=* 0.032).

**Figure 1.**
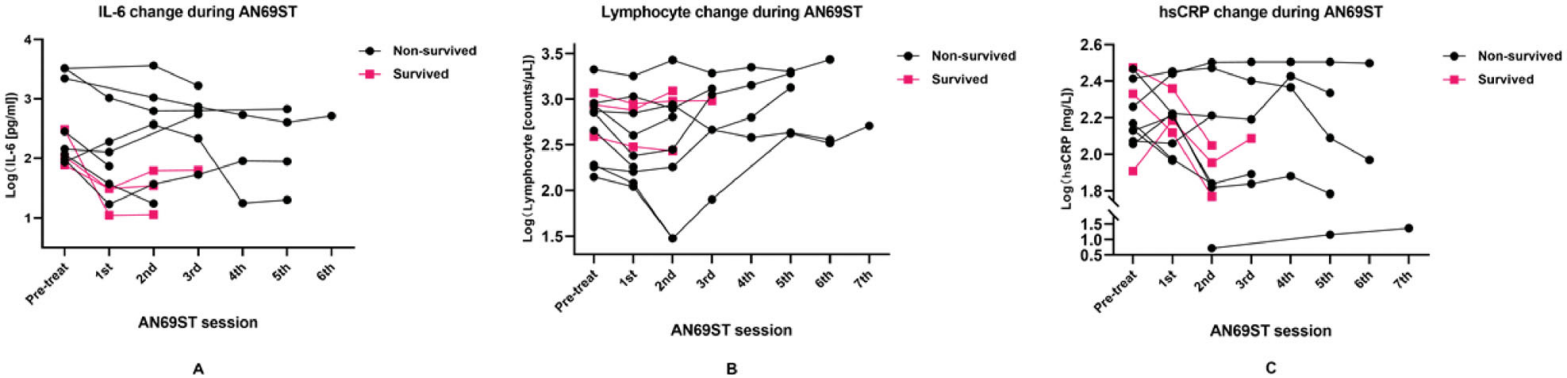
Changes during AN6ST. (A) IL-6 change during AN6ST. (B) Lymphocyte change during AN6ST. (C) hs-CRP change during AN6ST.

**Figure 2.**
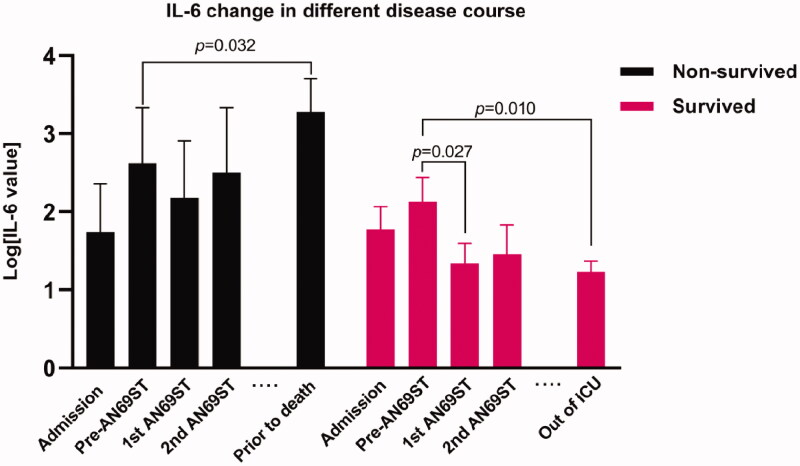
IL-6 change during different disease course.

Admittedly, the survival rate in our AN69ST group was not fairly promising. However, it was among the first blood purification attempts in treating critically severe COVID-19 patients globally, when there was little experience toward this unknown disease. One distinct finding surfaced from the current case series is that inflammatory factors associate disease severity; therefore, monitoring inflammation is essential, especially for critically severe COVID-19 patients. The lower initial inflammatory levels and earlier response toward IL-6 normalization may indicate a better prognosis, whereas the irreversible high-level IL-6, despite blood purification, results in consequent death. We want to share our opinion of “windows of opportunity” for severe COVID-19 patients. For patients who tend to develop into critically severe, the time left for intervention is limited. The average time to apply AN69ST in our patients might be too late when they developed significantly increased inflammatory levels [10]. Early application of blood purification with intensive dose in severe COVID-19 patients may achieve better efficacy and realize therapeutic goals such as stabilizing hemodynamics and improving MODS. AN69ST is a mature and relatively safe method ready to apply. We propose that multi-disciplinary effort is necessary to maximize the potentiality of blood purification for severe patients in earlier disease phases. Future studies should aim to design RCTs rationally to assess whether the application of AN69ST in critically ill COVID-19 patients improves the clinical prognosis, including prevention of organ failure, reduction in length of hospital stay, and improvement in survival. In addition, the negative impact of removing potentially beneficial molecules (e.g., protective antibodies) during blood purification should also be evaluated.

## Data Availability

All necessary data have been presented as tables and figures in the manuscript. Related information is accessible under request to the corresponding author.
